# Catecholamine modulation of frontal cortical ignition during wakefulness, sleep and anesthesia

**DOI:** 10.1093/nsr/nwag412

**Published:** 2026-07-06

**Authors:** Bing Li, Jia Zhao, Yang Dan

**Affiliations:** Department of Anesthesiology, Songjiang Research Institute; Department of Emergency and Critical Disease, Songjiang Research Institute, Shanghai Key Laboratory of Emotions and Affective Disorders, Songjiang Hospital Affiliated to Shanghai Jiao Tong University School of Medicine, Shanghai 201600, China; Department of Neuroscience, Helen Wills Neuroscience Institute, Howard Hughes Medical Institute, University of California, Berkeley, Berkeley, CA 94720, USA; Department of Anesthesiology, Songjiang Research Institute; Department of Emergency and Critical Disease, Songjiang Research Institute, Shanghai Key Laboratory of Emotions and Affective Disorders, Songjiang Hospital Affiliated to Shanghai Jiao Tong University School of Medicine, Shanghai 201600, China; Department of Neuroscience, Helen Wills Neuroscience Institute, Howard Hughes Medical Institute, University of California, Berkeley, Berkeley, CA 94720, USA

**Keywords:** dopamine, noradrenaline, anesthesia, sleep, conscious awareness

## Abstract

Conscious awareness—diminished during non-rapid-eye-movement (NREM) sleep and general anesthesia—is prevalent during wakefulness, but the underlying mechanism remains poorly understood. Here, we show that activity of catecholamine neurons, known to be higher during wakefulness than NREM sleep, enhances cortical ignition, a process closely associated with conscious awareness. Chemogenetic activation and inactivation of catecholamine neurons, particularly dopaminergic neurons in the ventral tegmental area (VTA), respectively, increased and decreased the amplitude of anterior cingulate area (ACA) excitation evoked by visual cortex (V1) stimulation. Ketamine and isoflurane, two commonly used general anesthetics, greatly reduced V1-to-ACA activity propagation. In addition to suppressing ongoing cortical activity, these general anesthetics significantly reduced the activity of VTA dopaminergic and basal forebrain cholinergic neurons, with dopaminergic activity dropping well below its level during NREM sleep. The strong inhibition of frontal cortical activity evoked by V1 stimulation, prominent during NREM sleep, was also absent under ketamine and isoflurane anesthesia. These results indicate that general anesthesia and NREM sleep suppress global ignition through overlapping but non-identical mechanisms.

## INTRODUCTION

Conscious awareness, a core aspect of subjective experience, is diminished during both non-rapid-eye-movement (NREM) sleep and general anesthesia [1–3]. Although sleep and anesthesia share many phenotypic similarities, there is ongoing debate about the extent to which they are mediated by common neuronal mechanisms [4–9]. Anesthetic drugs have been shown to activate sleep-promoting neurons in the hypothalamus [10–15], indicating shared circuits. On the other hand, lesion and manipulation experiments suggest that these anesthesia-activated, sleep-promoting neurons may not be necessary or sufficient for the induction of general anesthesia [16,17]. Since many anesthetic drugs act on transmitter receptors and ion channels that are widely expressed in the brain [5–7,18], they are likely to affect the activity of multiple cell types in multiple brain regions [3].

Conscious awareness of sensory stimuli is associated with the propagation of neuronal signals from the sensory cortex to parietal and frontal cortical areas, known as the ‘global neuronal workspace’ [19–22]. The amplification of sensory signals through recurrent excitation between cortical neurons, referred to as ‘global ignition’, is essential for this process. In a previous study, we found that the propagation of excitatory activity from the visual cortex (V1) to the anterior cingulate area (ACA) of the frontal cortex in mice is strongly suppressed during NREM sleep compared to wakefulness, due to a powerful inhibition of ACA pyramidal neurons evoked by V1 stimulation [23]. Whether and how general anesthesia affects the V1-to-ACA activity propagation remain unclear. Furthermore, chemogenetic manipulations showed that the basal forebrain (BF) neurons releasing acetylcholine (ACh), known to be much more active during wakefulness than NREM sleep [24,25], powerfully promote V1-to-ACA activity propagation [23]. How ACh neuron activity is modulated by general anesthesia is unclear. Besides ACh, neurons releasing catecholamines, including dopamine (DA) and norepinephrine (NE), also play crucial roles in promoting wakefulness and arousal [26–36]. Whether and how catecholamine neurons are affected by general anesthesia and how they, in turn, modulate global ignition remain unclear.

In this study, we used simultaneous optogenetic and chemogenetic manipulations and fiber photometry calcium imaging to examine the roles of DA and NE neurons in modulating global ignition during wakefulness, NREM sleep and anesthesia. We found that the ventral tegmental area (VTA) DA neuron activity significantly enhances cortical ignition, above and beyond its role in promoting wakefulness. Under ketamine- and isoflurane-induced general anesthesia, the excitatory response of ACA neurons to V1 stimulation was greatly reduced, but the inhibitory response that is prominent during NREM sleep was largely absent. In addition to suppressing ongoing cortical activity, these general anesthetics also suppressed both VTA DA and BF ACh neuron activity, with DA neuron activity dropping well below its level observed during NREM sleep. These results indicate that the suppression of cortical ignition during NREM sleep and general anesthesia is mediated by overlapping but non-identical mechanisms, and that DA signaling plays a powerful role in regulating cortical ignition across different brain states.

## RESULTS

**Figure 1.**
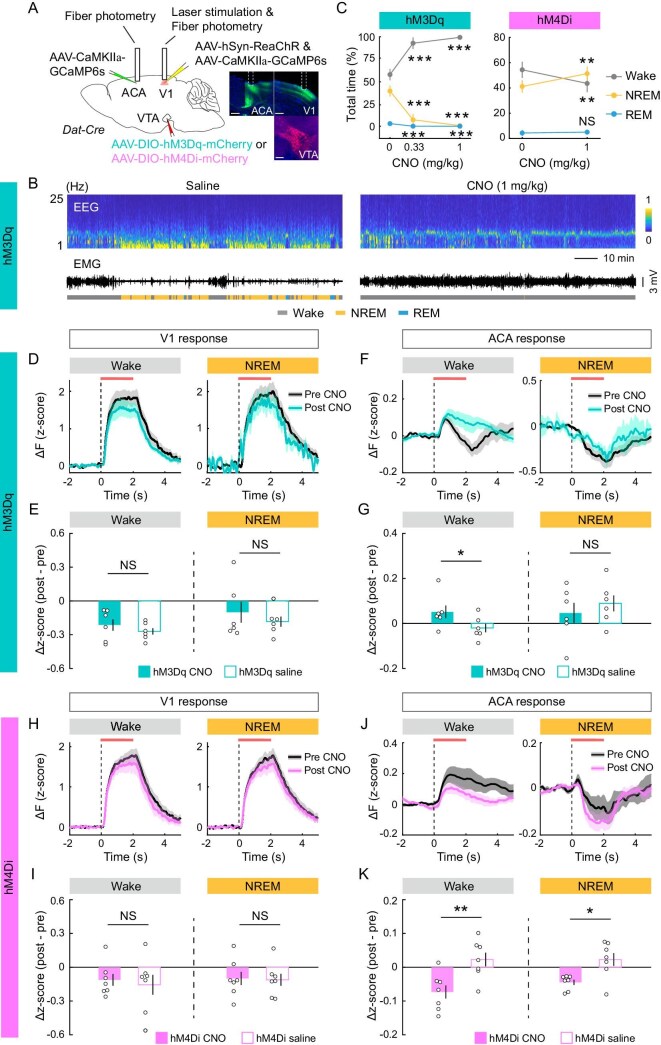
Effects of chemogenetic manipulation of VTA dopaminergic neurons on V1-to-ACA activity propagation. (A) Schematic of virus injection and fiber photometry imaging. GCaMP6s expression (green) in ACA and V1 pyramidal neurons, and mCherry expression (red) in VTA dopaminergic neurons, are shown on the right. Dashed lines, optic fiber tract. Scale bar, 500 μm for ACA and V1, 200 μm for VTA. (B) Example sessions illustrating EEG power spectrogram, EMG and color-coded brain states following saline or CNO (1 mg/kg) injection in mice expressing hM3Dq in VTA dopaminergic neurons. (C) Total time in wake, NREM or REM states following saline (0 mg/kg CNO) or CNO injections at different dosages in mice expressing hM3Dq (left) or hM4Di (right) in dopaminergic neurons. (Left) Wake: saline versus 0.33 mg/kg CNO, *P* = 6 × 10^−8^; saline versus 1 mg/kg CNO, *P* = 1.4 × 10^−8^. NREM: saline versus 0.33 mg/kg CNO, *P* = 1.9 × 10^−7^; saline versus 1 mg/kg CNO, *P* = 3.5 × 10^−8^. REM: saline versus 0.33 mg/kg CNO, *P* = 3.1 × 10^−6^; saline versus 1 mg/kg CNO, *P* = 4.5 × 10^−6^ [saline and 0.33 mg/kg CNO, *n* = 6 mice; 1 mg/kg CNO, *n* = 5 mice; one-way analysis of variance (ANOVA) with post-hoc Tukey’s tests]. (Right) Wake, *P* = 0.0042; NREM, *P* = 0.0061; REM, *P* = 0.26 (*n* = 7 mice, paired *t-*test). (D) Optogenetically evoked calcium activity in V1 pyramidal neurons before (black) and after (colored) CNO injection (0.33 mg/kg) during wakefulness (left) or NREM sleep (right), averaged across mice expressing hM3Dq in dopaminergic neurons. Baseline signal was subtracted for each trial before averaging. Horizontal red bar, laser stimulation. (E) Summary of amplitude difference in V1 before and after CNO (filled) or saline (open) injection. Each point represents one mouse. Wake, *P* = 0.4; NREM, *P* = 0.57 (*n* = 6 mice, paired *t*-test). (F and G) Similar to panels (D) and (E), but for calcium activity in ACA pyramidal neurons. In panel (G), wake, *P* = 0.028; NREM, *P* = 0.6. (H–K) Similar to panels (D) and (E), but for V1 and ACA responses averaged across mice expressing hM4Di in dopaminergic neurons (CNO, 1 mg/kg, *n* = 7 mice). In panel (I), wake, *P* = 0.62; NREM, *P* = 0.9. In panel (K), wake, *P* = 0.0038; NREM, *P* = 0.04. Bar and error bar, mean ± standard error of the mean (SEM). Shading, ±SEM. *P < 0.05; **P < 0.01; ***P < 0.001; NS, not significant.

A previous study showed that the calcium responses of ACA pyramidal neurons evoked by optogenetic activation of V1 neurons provide a robust measure of brain-state-dependent activity propagation between sensory and frontal cortical areas [23]. In this study, we used the same paradigm to examine the effects of catecholamine modulation and general anesthesia on cortical ignition. We injected AAV expressing ReaChR [37] (AAV2-hSyn-ReaChR-mCitrine) into V1 and AAV9-CaMKIIα-GCaMP6s into both V1 and ACA of mice to target pyramidal neurons (Fig. 1A). A brief laser stimulation (635 nm, 2–4 mW, 2 s) was applied in V1, and fiber photometry was used to measure V1 and ACA calcium responses in freely moving mice (∼180 trials/session, 2–3 sessions/mouse). Wake, rapid-eye-movement (REM) and NREM sleep were classified based on electroencephalogram (EEG) and electromyogram (EMG) recordings.

### Effects of catecholamine neuron activity on cortical ignition

To test the effect of midbrain DA neuron activation on V1-to-ACA activity propagation, we injected AAV2-DIO-hM3Dq-mCherry into the VTA of *Dat-Cre* mice (Fig. 1A). Intraperitoneal (IP) injection of CNO at 1 mg/kg, a commonly used dosage for chemogenetic activation, induced persistent wakefulness (∼100%) over the following 2 h (Fig. 1B and C), which precludes the measurement of V1-to-ACA activity propagation during sleep. Thus, for the DA neuron activation experiment, we reduced the CNO level to 0.33 mg/kg, which still increased wakefulness and decreased both REM and NREM sleep (Fig. 1C) but allowed some test trials during NREM sleep. Despite the powerful wake-promoting effect, DA neuron activation did not change the EEG power spectrum significantly within each brain state (Fig. S1A and B), distinct from the effect of activating BF ACh neurons [23].

We then compared V1-evoked calcium responses in both V1 and ACA before and after CNO injection within the same brain state. We found no significant effect on laser-induced activation of V1 neurons (Fig. 1D and E, and Fig. S1C), likely because V1 activity was dominated by direct optogenetic activation. In contrast, CNO injection caused a significant increase in ACA response during wakefulness but not during NREM sleep (Fig. 1F). In the control experiment, saline injection caused no significant change in the calcium responses (Fig. S1D), and the difference between the responses before and after injection was significantly larger for CNO than saline during wakefulness (Fig. 1G). These results indicate that VTA DA neuron activation enhances activity propagation from V1 to the frontal region ACA.

We next tested the effect of DA neuron inactivation by injecting AAV2-DIO-hM4Di-mCherry into the VTA of *Dat-Cre* mice (Fig. 1A). CNO injection (1 mg/kg) had no significant effect on EEG power spectrum and laser-induced activation of V1 neurons (Fig. 1H and I, and Fig. S1E–G), but caused significant decreases in ACA calcium responses during both NREM sleep and wakefulness (Fig. 1J and K, and Fig. S1H) as well as decreased time in wakefulness (Fig. 1C), opposite to the effects of hM3Dq-mediated DA neuron activation.

**Figure 2.**
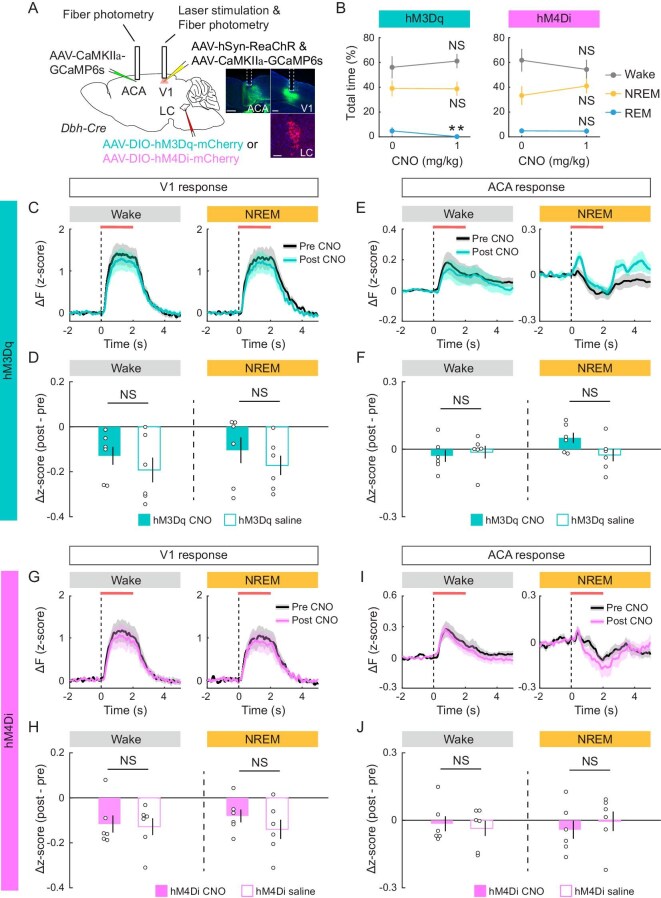
Effects of chemogenetic manipulation of LC noradrenergic neurons on V1-to-ACA activity propagation. (A) Schematic of virus injection and fiber photometry imaging. GCaMP6s expression (green) in ACA and V1 pyramidal neurons, and mCherry expression (red) in LC noradrenergic neurons, are shown on the right. Dashed lines, optic fiber tract. Scale bar, 500 μm for ACA, 200 μm for V1 and LC. (B) Total time in wake, NREM or REM states following saline (0 mg/kg CNO) or CNO injection in mice expressing hM3Dq (left, *n* = 6 mice) or hM4Di (right, *n* = 6 mice) in noradrenergic neurons. (Left) Wake, *P* = 0.38; NREM, *P* = 0.93; REM, *P* = 0.0087. (Right) Wake, *P* = 0.14; NREM, *P* = 0.077; REM, *P* = 0.85 (paired *t-*test). (C) Optogenetically evoked calcium activity in V1 pyramidal neurons before (black) and after (colored) CNO injection (1 mg/kg) during wakefulness (left) or NREM sleep (right), averaged across mice expressing hM3Dq in noradrenergic neurons. Baseline signal was subtracted for each trial before averaging. Horizontal red bar, laser stimulation. (D) Summary of amplitude difference in V1 before and after CNO (filled) or saline (open) injection. Each point represents one mouse. Wake, *P* = 0.1; NREM, *P* = 0.23 (*n* = 6 mice, paired *t*-test). (E and F) Similar to panels (C) and (D), but for calcium activity in ACA pyramidal neurons. In panel (F), wake, *P* = 0.66; NREM, *P* = 0.1. (G–J) Similar to panels (C)–(F), but for V1 and ACA responses averaged across mice expressing hM4Di in noradrenergic neurons (*n* = 6 mice). In panel (H), wake, *P* = 0.76; NREM, *P* = 0.32. In panel (J), wake, *P* = 0.59; NREM, *P* = 0.57. Bar and error bar, mean ± SEM. Shading, ±SEM. *P < 0.05; **P < 0.01; ***P < 0.001; NS, not significant.

Besides DA neurons, other catecholamine-releasing neurons, especially the NE neurons in the locus coeruleus (LC), are also known to promote arousal [26–28]. To test the role of LC NE neurons in regulating cortical ignition, we injected AAV2-DIO-hM3Dq-mCherry or AAV2-DIO-hM4Di-mCherry into the LC of *Dbh-Cre* mice (Fig. 2A). Unlike VTA DA neurons, activation of LC NE neurons with 1 mg/kg of CNO (IP) caused only a suppression of REM sleep but no significant change in wakefulness or NREM sleep (Fig. 2B). NE neuron activation also caused no significant change in laser-evoked V1 or ACA response (Fig. 2C–F, and Fig. S2A and B), although during NREM sleep there appeared to be a slight (not statistically significant) increase in the transient excitation of ACA neurons (Fig. 2E). Chemogenetic inactivation of LC NE neurons (1 mg/kg CNO) also had no significant effect on sleep-wake brain states (Fig. 2B) or laser evoked V1 or ACA responses (Fig. 2G–J, and Fig. S2C and D). Thus, compared to VTA DA neurons, LC NE neurons have a much weaker effect on brain states or cortical ignition, at least under the current experimental conditions.

### Effects of ketamine and isoflurane anesthesia on cortical ignition

Conscious awareness is also diminished during general anesthesia. We next measured how ketamine and isoflurane, two commonly used general anesthetics, affect cortical ignition. In each imaging session, we first measured the V1-to-ACA activity propagation across natural sleep-wake cycles for ∼1 h; ketamine or isoflurane was then administered, and the calcium responses to V1 stimulation were measured under general anesthesia.

**Figure 3.**
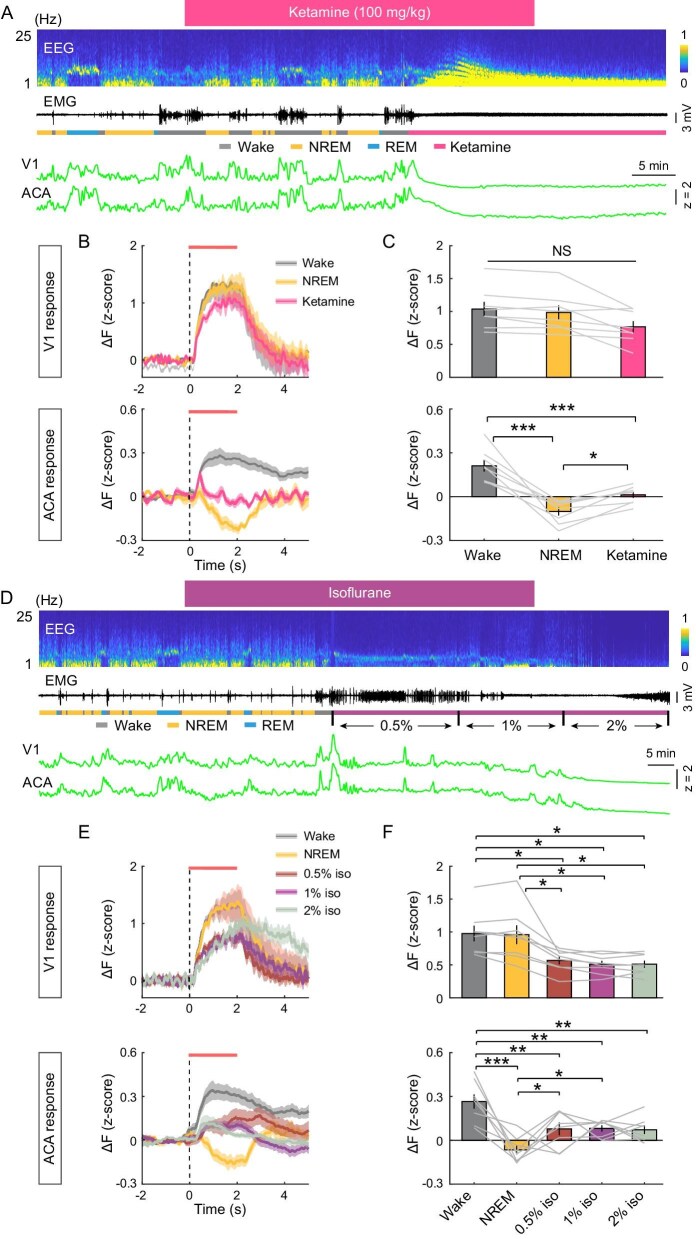
Effects of general anesthesia (ketamine or isoflurane) on V1-to-ACA activity propagation. (A) Example session showing EEG, EMG and calcium activity in V1 and ACA pyramidal neurons before and after ketamine injection. (B) Optogenetically evoked calcium activity in V1 (top) or ACA pyramidal neurons (bottom), averaged across mice. Baseline signal was subtracted for each trial before averaging. Horizontal red bar, laser stimulation. (C) Differences in averaged response amplitudes in V1 (top) and ACA (bottom) during wakefulness, NREM sleep and ketamine application for each mouse. For V1 (top), no significant difference was found among different brain states. For ACA (bottom): wake versus NREM, *P* = 1.1 × 10^−6^; wake versus ketamine, *P* = 4.2 × 10^−4^; NREM versus ketamine, *P* = 0.039 (*n* = 8 mice, one-way ANOVA with post-hoc Tukey’s). (D–F) Similar to panels (A–C), but with isoflurane (iso) used for general anesthesia (*n* = 8 mice). In panel (F), for V1 (top), wake versus 0.5% iso, *P* = 0.035, wake versus 1% iso, *P* = 0.013, wake versus 2% iso, *P* = 0.014, NREM versus 0.5% iso, *P* = 0.046, NREM versus 1% iso, *P* = 0.017, NREM versus 2% iso, *P* = 0.019; for ACA (bottom), wake versus NREM, *P* = 1.7 × 10^−6^, wake versus 0.5% iso, *P* = 0.0077, wake versus 1% iso, *P* = 0.0083, wake versus 2% iso, *P* = 0.0047, NREM versus 0.5% iso, *P* = 0.05, NREM versus 1% iso, *P* = 0.047. Bar and error bar, mean ± SEM. Shading, ±SEM. *P < 0.05; **P < 0.01; ***P < 0.001; NS, not significant.

As shown in Fig. 3A, ketamine injection (IP, 100 mg/kg, together with 10 mg/kg xylazine) caused a rapid change in the EEG spectrogram and a reduction in EMG activity relative to wakefulness. Within 1–2 min of ketamine/xylazine injection, the EEG power spectrum exhibited multiple equally spaced harmonic bands, indicating a strong oscillation at 2–3 Hz. This may reflect the rhythmic activity in the retrosplenial cortex induced by a subanesthetic dose of ketamine [38]. After ∼5 min, the harmonic bands were replaced by broadband low-frequency (<5 Hz) signals, which persisted through the 25-min imaging period. Before anesthesia, the ongoing calcium activity in both V1 and ACA pyramidal neurons was significantly lower during NREM sleep than wakefulness and REM sleep. Ketamine/xylazine injection caused a marked reduction of ongoing activity in both cortical areas, below the levels observed during NREM sleep.

We next analyzed the calcium responses evoked by optogenetic stimulation in V1. Whereas ketamine caused only a slight reduction in V1 response, which was statistically not significant, the response in the ACA was markedly lower in amplitude and shorter in duration (Fig. 3B and C). This indicates that ketamine anesthesia suppresses V1-to-ACA activity propagation, consistent with ketamine-induced breakdown of functional connectivity between cortical areas measured by EEG and functional magnetic resonance imaging (fMRI) in humans and non-human primates [39–42]. Notably, although V1-evoked ACA excitation is suppressed by both NREM sleep and ketamine anesthesia, the effects are distinct; the strong V1-evoked inhibition of ACA observed during NREM sleep is absent under ketamine anesthesia [23].

We then tested the effect of isoflurane anesthesia. The ongoing activity of V1 and ACA pyramidal neurons showed a progressive decrease with increasing levels of isoflurane (Fig. 3D). Laser-evoked responses were significantly reduced in both V1 and ACA, although the reduction in ACA was stronger than the reduction in V1 (58%, 52% and 52% of the amplitude measured during wakefulness for V1, under 0.5%, 1% and 2% isoflurane, respectively; 30%, 30% and 26% for ACA, respectively; Fig. 3E and F). The inhibitory response in the ACA observed during NREM sleep was also absent under isoflurane anesthesia.

### Effects of ketamine and isoflurane on DA and ACh neuron activity

Having shown that V1-to-ACA activity propagation is strongly modulated by both VTA DA neurons (Fig. 1) and BF ACh neurons [23], we wondered whether the suppression of activity propagation by general anesthesia (Fig. 3) is partly attributable to changes in the activity of these neuromodulatory circuits. To measure the activity of DA and ACh neurons, we injected AAV9-Flex-GCaMP6s into the VTA of *Dat-Cre* or BF of *Chat-Cre* mice and used fiber photometry to measure the calcium signals across sleep–wake cycles and under general anesthesia.

**Figure 4.**
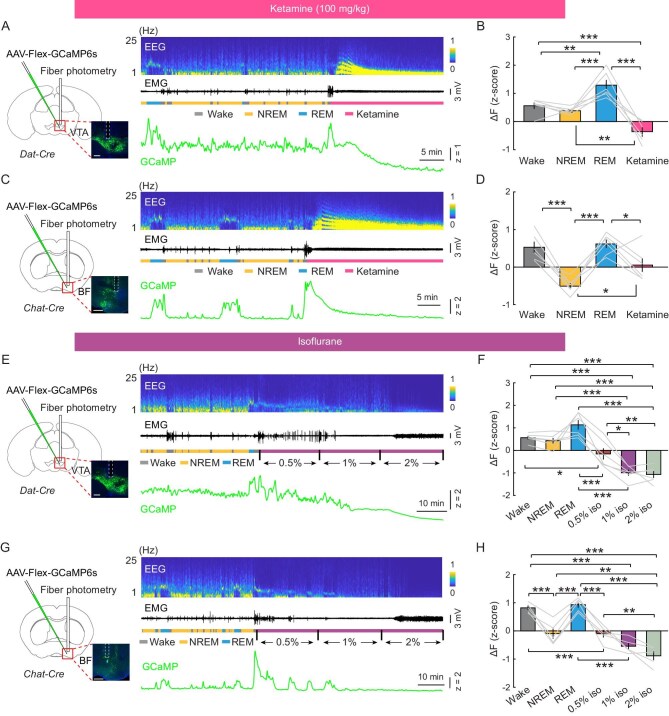
Effects of general anesthesia (ketamine or isoflurane) on calcium activity in VTA dopaminergic neurons and BF cholinergic neurons. (A) Schematic of virus injection and fiber photometry imaging (left), and an example session showing EEG, EMG and calcium activity in VTA dopaminergic neurons before and after ketamine injection (right). The inset shows GCaMP6s expression (green) in VTA dopaminergic neurons. Dashed white lines, optic fiber tract. Scale bar, 200 μm. (B) Mean calcium activity in VTA dopaminergic neurons across different brain states. Wake versus REM, *P* = 0.0054; wake vs. ketamine, *P* = 5.7 × 10^−4^; NREM versus REM, *P* = 6.6 × 10^−4^; NREM versus ketamine, *P* = 0.0047; REM versus ketamine, *P* = 2 × 10^−7^ (*n* = 6 mice, one-way ANOVA with post-hoc Tukey’s). (C and D) Similar to panels (A and B), but for calcium activity in BF cholinergic neurons (*n* = 6 mice). In panel (C), the inset shows GCaMP6s expression (green) in BF cholinergic neurons. Dashed white lines, optic fiber tract. Scale bar, 500 μm. In panel (D), wake versus NREM, *P* = 1.5 × 10^−4^; NREM versus REM, *P* = 5.5 × 10^−5^; NREM versus ketamine, *P* = 0.038; REM versus ketamine, *P* = 0.038. (E–H) Similar to panels (A–D), but with isoflurane used for general anesthesia. In panel (F), wake versus 0.5% iso, *P* = 0.05; wake versus 1% iso, *P* = 4.2 × 10^−6^; wake versus 2% iso, *P* = 1.7 × 10^−6^; NREM versus 1% iso, *P* = 1.9 × 10^−5^; NREM versus 2% iso, *P* = 7.8 × 10^−6^; REM versus 0.5% iso, *P* = 9.0 × 10^−5^; REM versus 1% iso, *P* = 7.8 × 10^−9^; REM versus 2% iso, *P* = 3.5 × 10^−9^; 0.5% iso versus 1% iso, *P* = 0.016; 0.5% iso versus 2% iso, *P* = 0.0068; *n* = 6 mice. In panel (H), wake versus NREM, *P* = 6.3 × 10^−4^; wake versus 0.5% iso, *P* = 6.0 × 10^−4^; wake versus 1% iso, *P* = 2.02 × 10^−6^; wake versus 2% iso, *P* = 3.8 × 10^−8^; NREM versus REM, *P* = 1.4 × 10^−4^; NREM versus 2% iso, *P* = 0.0036; REM versus 0.5% iso, *P* = 1.4 × 10^−4^; REM versus 1% iso, *P* = 5.3 × 10^−7^; REM versus 2% iso, *P* = 1.1 × 10^−8^; 0.5% iso versus 2% iso, *P* = 0.0038; *n* = 5 mice. Bar and error bar, mean ± SEM. *P < 0.05; **P < 0.01; ***P < 0.001.

Imaging from the VTA in *Dat-Cre* mice showed that DA neurons are more active during wakefulness and REM sleep than NREM sleep, consistent with previous studies [30]. Ketamine/xylazine injection rapidly and strongly reduced DA neuron activity, significantly below the level observed during NREM sleep (Fig. 4A and B). BF ACh neurons were also more active during wakefulness and REM sleep than NREM sleep (Fig. 4C). Although the absolute firing rate cannot be inferred from fiber photometry calcium imaging, previous studies using optrode recordings showed that BF ACh neuron firing rates during NREM sleep were close to 0 [24,25]. Ketamine/xylazine injection caused an initial surge in ACh neuron activity, followed by a gradual decrease, approaching the level during NREM sleep after ∼10 min. Thus, ketamine anesthesia suppressed the activity of both DA (Fig. 4B) and ACh neurons (Fig. 4D), but with different time courses.

We also tested the effects of isoflurane anesthesia. Isoflurane at 1% or 2% strongly suppressed the activity of both DA and ACh neurons (Fig. 4E–H). Thus, both ketamine and isoflurane strongly suppress the activity of these arousal-promoting neuromodulatory systems, which is likely to contribute to the suppression of cortical ignition under general anesthesia (Fig. 3).

## DISCUSSION

In this study, we characterized the modulation of cortical ignition by two wake-promoting catecholamines, DA and NE, and compared the effects of general anesthesia and natural sleep. Using simultaneous optogenetic and chemogenetic manipulations along with fiber photometry measurements, we showed that VTA DA neurons, but not LC NE neurons, powerfully promote wakefulness and enhance V1-to-ACA activity propagation (Figs 1 and 2). Although in principle, the lack of effect of LC NE neurons could be due to insufficient chemogenetic activation, this seems unlikely since the CNO dose used (1 mg/kg) was higher than that used for VTA neuron activation (0.33 mg/kg), and it completely suppressed REM sleep (Fig. 2B). Instead, the difference is likely at the circuit level: whereas LC NE neurons are known to improve signal-to-noise ratios [43,44] through diffuse projections, VTA DA neurons may amplify signal propagation by modulating the striatal–thalamic–cortical loop. Together with BF ACh neurons, which also enhance frontal cortical ignition [23], VTA DA neurons are strongly suppressed by ketamine and isoflurane anesthesia (Fig. 4), which is likely to contribute to anesthesia-induced reduction of cortical ignition (Fig. 3 and Fig. S4). Interestingly, the effect of VTA DA neuron activity on cortical ignition depends on the brain state: their chemogenetic activation increased V1-evoked ACA response during wakefulness but not during NREM sleep (Fig. 1F and G). In addition to BF cholinergic neurons, the ongoing activity of excitatory and inhibitory cortical neurons are also different between NREM sleep and wakefulness, which could contribute to the state dependence of the effect.

The mechanism by which general anesthetics induce the loss of consciousness has been investigated for decades. Ketamine preferentially suppresses N-methyl-D-aspartate (NMDA) receptor-mediated glutamatergic inputs to gamma-aminobutyric acid (GABA)-ergic interneurons, resulting in abnormal excitatory activity in the cortex and other brain regions [7]; it may also affect brain activity by potentiating μ-opioid receptor signaling [45]. In contrast, isoflurane acts primarily through potentiation of GABA_A_ receptor-mediated inhibition [6]. Although great progress has been made in identifying these drug targets at the molecular level, the underlying neuronal circuits remain incompletely understood [5–7]. We found that both ketamine and isoflurane strongly suppressed V1-to-ACA activity propagation in mice (Fig. 3), reminiscent of previous findings in humans that functional connectivity between cortical areas is reduced under anesthesia [39–42,46,47]. Furthermore, these anesthetics strongly suppressed VTA DA neurons and BF ACh neurons (Fig. 4), whose activity is also reduced during NREM sleep. These results are consistent with the idea that the loss of consciousness during natural sleep and general anesthesia share some common neuronal mechanisms [8,10–15].

Our experiments also point to important differences between sleep and anesthesia. In particular, the strong inhibitory response of ACA neurons to V1 stimulation was observed during NREM sleep [23] but not under ketamine or isoflurane anesthesia (Fig. 3B, C, E and F). At an anesthetic dose of ketamine (100 mg/kg) with xylazine, the activity of cortical pyramidal neurons and VTA DA neurons was also significantly lower than the levels during NREM sleep (Fig. S3B and C). Interestingly, at 5–10 min after ketamine/xylazine injection, BF ACh neurons were still highly active (Fig. S3A), suggesting that the reduction of V1-evoked ACA response during this period (Fig. S3D) was not due to diminished BF ACh neuron activity, different from that during NREM sleep [23]. On the other hand, previous studies showed that DA could decrease somatic inhibition and increase dendritic inhibition [48]. The strong decline in VTA DA neuron activity during this period may result in increased somatic inhibition, which is particularly effective in suppressing the spiking output of pyramidal neurons [49], contributing to the reduction of V1-evoked ACA response. Indeed, the suppression of cortical ignition induced by anesthesia could be partially restored by chemogenetic activation of DA neurons (Fig. S4). In addition to the differences in neuromodulator levels, the lack of V1-evoked inhibition of ACA pyramidal neurons under ketamine or isoflurane anesthesia also suggests that activity patterns of cortical inhibitory interneurons are distinct from those during NREM sleep [23].

The suppression of cortical pyramidal neurons and subcortical neuromodulatory systems could be caused by an overall enhancement of GABAergic inhibition in the brain induced by the general anesthetics [5–7]. In the case of DA neurons, previous studies have shown that isoflurane and propofol can activate the lateral habenula [50,51], which is known to excite the GABAergic neurons that in turn inhibit VTA DA neurons [52]. Electrical or optogenetic activation of DA neurons has been shown to cause rapid awakening from general anesthesia [34–36]. In our study, chemogenetic activation of VTA DA neuron promoted wakefulness more powerfully than NE and ACh neurons [23] (Figs 1 and 2), and it enhanced cortical ignition not only during wakefulness but also under anesthesia (Fig. S4). Together, these results support an essential role of DA signaling in sustaining wakefulness and conscious awareness [33].

### Limitations

While the current study examined the roles of catecholamine neurons in regulating cortical ignition under wakefulness, sleep and anesthesia, much remains to be investigated. In the experiments with isoflurane anesthesia, we used sequential dosing (0.5%, 1% and then 2%). Although each concentration was maintained for ∼20 min, the observed effects at higher concentrations may partially reflect the residual effects from prior exposures, limiting our ability to isolate pure concentration-dependent effects. Different anesthetics are known to induce the loss of consciousness through diverse mechanisms [7]. While the current study examined ketamine/xylazine and isoflurane, whether other anesthetics (e.g. dexmedetomidine or propofol) exert similar or different effects on cortical ignition and catecholamine neuron activity remains to be investigated. In addition to ACh, DA and NE, other wake-promoting neuromodulatory systems such as serotonin, histamine and orexin are also likely affected by general anesthesia [6,53]. Furthermore, anesthesia can suppress inputs from modulatory thalamic nuclei to the cortex [54–58], known to play a crucial role in enabling consciousness [59–61]. It would be of great interest in future studies to examine how these additional pathways contribute to the regulation of cortical ignition.

## MATERIALS AND METHODS

All procedures were approved by Animal Care and Use Committees of the University of California, Berkeley (AUP-2016-06-8860). Detailed materials and methods are available in the Supplementary data.

## Supplementary Material

nwag412_Supplemental_File

## Data Availability

All relevant data are included in the article and in the Supplementary data.
