# Supplementary material for: Catecholamine modulation of frontal cortical ignition during wakefulness, sleep and anesthesia
Source: Natl Sci Rev. 2026 Jul 6;13(15):nwag412. doi: 10.1093/nsr/nwag412 (PMC13463767; doi:10.1093/nsr/nwag412)
Supplement: Supplementary file 1 [file nwag412_supplemental_file.pdf]

Supporting Information for

Catecholamine modulation of frontal cortical ignition during wakefulness, sleep, and anesthesia

Bing Li[1,2,†], Jia Zhao[1,†] and Yang Dan[2,††,*]

[1]Department of Anesthesiology, Songjiang Research Institute, Shanghai Key Laboratory of Emotions and Affective Disorders, Songjiang Hospital Affiliated to Shanghai Jiao Tong University School of Medicine, Shanghai 201600, China; Department of Emergency and Critical Disease, Songjiang Research Institute, Shanghai Key Laboratory of Emotions and Affective Disorders, Songjiang Hospital Affiliated to Shanghai Jiao Tong University School of Medicine, Shanghai 201600, China

[2]Department of Neuroscience, Helen Wills Neuroscience Institute, Howard Hughes Medical Institute, University of California, Berkeley, Berkeley, CA 94720, USA

[††]Present address: Institute of Neuromodulation and Cognition (INC), Shenzhen Medical Academy of Research and Translation (SMART), Shenzhen, Guangdong 518107, China

[†]Contributed equally to this work.

[*]**Corresponding author.** Email: ydan@berkeley.edu

This PDF file includes:

Materials and methods

Figs. S1 to S4

MATERIALS AND METHODS

Animals

C57BL/6J (000664), *Chat-Cre* (006410), *Dat-Cre* (006660), and *Dbh-Cre* (033951) mice were obtained from The Jackson Laboratory. All experiments were conducted on adult mice (2−6 months) of both genders. Mice of specific genotypes were randomly assigned to experimental and control groups and underwent identical surgical and behavioral procedures. Results from male and female mice showed no significant difference, so the data were combined. Mice were housed under a 12 h light−dark cycle (lights on at 07:00 am and off at 19:00 pm) with ad libitum access to food and water. Mice with implants were housed individually. All procedures were approved by Animal Care and Use Committees of the University of California, Berkeley (AUP-2016-06-8860).

Virus preparation

AAV9-CAG-Flex-GCaMP6s (Addgene 100842-AAV9), AAV9-CaMKIIα-GCaMP6s (Addgene 107790-AAV9), AAV2-hSyn-DIO-hM3D(Gq)-mCherry (Addgene 44361-AAV2) and AAV2-hSyn-DIO-hM4D(Gi)-mCherry (Addgene 44362-AAV2) were obtained from the Addgene. AAV2-hSyn-ReaChR-mCitrine (Addgene, plasmid 50954) was prepared in-house following previously described protocols [1]. The final titer of all the viruses was estimated to be $\geq 1 \times 10^{13}$ gc/ml.

Surgery

For all surgeries, mice were anesthetized with 1.5%−2% isoflurane and positioned on a stereotaxic frame. A heating pad was used to maintain body temperature throughout the

procedure, and eye ointment was applied to prevent drying. After shaving hairs and asepsis with Betadine and medical alcohol, an incision was made in the skin to expose the skull.

For virus injection experiments, a craniotomy (0.5−1 mm diameter) was made on top of the target region (coordinates provided below), and 0.1−0.3 μl virus was injected into the target region using Nanoject II (Drummond Scientific) with a micropipette. For chemogenetic manipulation of VTA dopaminergic neurons and LC noradrenergic neurons, AAV2-hSyn-DIO-hM3D(Gq)-mCherry or AAV2-hSyn-DIO-hM4D(Gi)-mCherry was bilaterally injected into the VTA or LC.

For sleep-wake recording, EEG and EMG electrodes were implanted. EEG recordings were obtained from two stainless steel screws on top of the cortex: one at anteroposterior (AP) −3 mm, mediolateral (ML) 2.5 mm and the other at AP +1.5 mm, ML 1.5 mm. Two EMG electrodes were inserted into the neck musculature. A reference screw was positioned in the skull over the left cerebellum. Insulated leads from the EEG and EMG electrodes were soldered to a pin header, which was then secured to the skull with dental cement.

For fiber photometry imaging and optogenetic activation experiments, a craniotomy (0.5−1 mm diameter) was performed on top of the target region (coordinates provided below), and optical fibers (Neurophotometrics, 200 μm core, 0.37 NA) with FC ferrules were implanted approximately 100 μm above the target region. Dental cement was applied to cover the exposed skull completely and to secure the EEG and EMG recording implants to the screws.

After surgery, mice were allowed to recover from anesthesia on a heating pad before being returned to their home cage. Buprenorphine (0.1 mg/kg) was administered prior to surgery, and meloxicam (5–10 mg/kg) was given prior to surgery and again the following day. All experiments were conducted at least one week after surgery.

Stereotaxic coordinates for virus injection and optic fiber implantation:

V1: AP -3.1 mm, ML 2.5 mm, DV 0.5 mm.

ACA: AP 0.5 mm, ML 0.3 mm, DV 0.7 mm.

VTA: AP -3.5 mm, ML 0.5 mm, DV 4.4 mm.

LC: AP -5.5 mm, ML 0.9 mm, DV 4 mm.

Basal forebrain: AP 0.1 mm, ML 1.5 mm, DV 5.3 mm.

Sleep-wake recording and analysis

Experiments were conducted in sound-attenuating boxes between 10:00 am and 19:00 pm. EEG and EMG electrodes were connected to flexible recording cables via a mini-connector. Signals were recorded using a Tucker-Davis Technologies (TDT) PZ5 amplifier, filtered (0–300 Hz) and digitized at 1,500 Hz. Spectral analysis was performed using fast Fourier transform, and brain states were classified into wake, NREM and REM states (wake: desynchronized EEG and high EMG activity; NREM: synchronized EEG with high-amplitude, low-frequency (1–4 Hz) activity and low EMG activity; REM: high EEG power at theta frequencies (6–9 Hz) and low EMG activity). Classification was performed in 5-s bins using a custom graphical user interface programmed in MATLAB (MathWorks).

Fiber photometry imaging and optogenetic activation

To record neuronal calcium activity in the VTA or BF, fiber photometry was performed using a TDT RZ10x real-time processor as previously described [2]. Mice, housed in their home cages within sound-attenuating boxes, were attached via an optical fiber (Neurophotometrics, 200 μm core, 0.37 NA) implanted in the VTA or BF to a fluorescence minicube (Doric Lenses) during imaging. Fluorescence elicited by 405 nm (serves as an isosbestic wavelength for GCaMP6s) and 465 nm (calcium-dependent GCaMP6s fluorescence) LEDs was filtered through the Doric minicubes and collected by an integrated photosensor on the RZ10x. Signals were pre-processed using TDT Synapse software and collected at a sampling frequency of 1,018 Hz.

For experiments involving both ACA and V1 fiber photometry imaging and/or optogenetic V1 activation, an optical fiber implanted in the ACA was connected to a Doric minicube for fiber photometry imaging, and another optic fiber implanted in the V1 was coupled to a minicube-wavelength division (Doric Lenses), which combined light from a fiber-coupled LED (for photometry) and a 635 nm laser (Laserglow, D631001FL, for optogenetic stimulation) into a single beam, then delivered to the brain via the fiber-optic implant [3]. Laser pulses (constant light, 2–4 mW at the fiber tip, 2 s) were generated using TDT Synapse software, with inter-trial intervals randomly set between 60 and 100 s.

Chemogenetic manipulation

Saline (0.9% NaCl) or clozapine N–oxide (CNO, Sigma, C0832, 1 mg/kg) was intraperitoneally injected into mice expressing hM3Dq-mCherry or hM4Di-mCherry in the VTA or LC. To avoid excessive wakefulness caused by activating VTA dopaminergic

neurons, CNO was diluted threefold before injection into mice expressing hM3Dq-mCherry in the VTA. To investigate V1-to-ACA activity propagation, fiber photometry imaging was performed for 2 h baseline period before saline or CNO injection and another 2 h afterwards. Each mouse was imaged for 4–6 sessions, with CNO administered randomly in half of the sessions and saline in the other half, maintaining the same circadian timing on different days.

Anesthesia experiment

The effects of general anesthetics were tested following ~1 h of recording across natural sleep-wake cycles in the home cage (see above). For ketamine-induced anesthesia, mice were injected with ketamine (100 mg/kg) and xylazine (10 mg/kg), a common veterinary protocol for inducing general anesthesia [4-6], and then returned to the home cage for further recording. For isoflurane-induced anesthesia, mice were transported in a recording chamber connected to a standard isoflurane vaporizer, and 0.5%, 1%, and 2% isoflurane was introduced sequentially, with each concentration administered for ~ 20 min. To test the effect of VTA DA neuron activation on cortical ignition during isoflurane-induced anesthesia, saline or CNO was injected at the beginning of anesthesia.

Fiber photometry data analysis

The 405-nm channel was used to correct for nonspecific, calcium-independent fluorescence changes, such as movement artifacts. For chemogenetic manipulation experiments, each channel (465-nm and 405-nm) was first fitted with a single exponential to remove the baseline change due to bleaching. The 405-nm signal was then fitted to the 465-nm signal using a least-squares linear fit method [7] and subtracted from the 465-nm

signal. For the anesthesia experiments, linear fitting was applied to the residuals to correct for bleaching. The resulting signals were converted to z-scores based on the mean and standard deviation of the entire imaging session. Response amplitude was computed as the average between 0 and 2 s after laser onset after subtracting the baseline period (0 to 2 s before laser onset).

Histology

Mice were deeply anesthetized and transcardially perfused with PBS, followed by 4% paraformaldehyde in PBS. Brains were post–fixed in 4% paraformaldehyde for 24–48 h and then stored in 30% sucrose in PBS for 48 h for cryoprotection. After embedding and freezing, brains were sectioned into 50 μm coronal slices using a cryostat (Leica). Fluorescence images were captured with a high-throughput slide scanner (Hamamatsu, Nanozoomer 2.0–RS) and a fluorescence microscope (Keyence, BZ–X710).

Statistical analysis

For statistical analyses, each mouse was treated as an individual unit. Analyses were conducted using MATLAB. Detailed statistical procedures are provided in the main text and figure legends.

REFERENCES

1. Hauswirth WW, Lewin AS, Zolotukhin S *et al.* Production and purification of recombinant adeno-associated virus. *Methods Enzymol*. 2000; **316**: 743-761. doi: 10.1016/s0076-6879(00)16760-6

2. Li B, Ma C, Huang YA *et al.* Circuit mechanism for suppression of frontal cortical ignition during NREM sleep. *Cell*. 2023; **186**(26): 5739-5750 e5717. doi: 10.1016/j.cell.2023.11.012

3. Silverman D, Chen C, Chang S *et al.* Activation of locus coeruleus noradrenergic neurons rapidly drives homeostatic sleep pressure. *Sci Adv*. 2025; **11**(3): eadq0651. doi: 10.1126/sciadv.adq0651

4. Bharioke A, Munz M, Brignall A *et al.* General anesthesia globally synchronizes activity selectively in layer 5 cortical pyramidal neurons. *Neuron*. 2022; **110**(12): 2024-2040 e2010. doi: 10.1016/j.neuron.2022.03.032

5. Hua T, Chen B, Lu D *et al.* General anesthetics activate a potent central pain-suppression circuit in the amygdala. *Nat Neurosci*. 2020; **23**(7): 854-868. doi: 10.1038/s41593-020-0632-8

6. Jiang-Xie LF, Yin L, Zhao S *et al.* A common neuroendocrine substrate for diverse general anesthetics and sleep. *Neuron*. 2019; **102**(5): 1053-1065 e1054. doi: 10.1016/j.neuron.2019.03.033

7. Lerner TN, Shilyansky C, Davidson TJ *et al.* Intact-brain analyses reveal distinct information carried by SNc dopamine subcircuits. *Cell*. 2015; **162**(3): 635-647. doi: 10.1016/j.cell.2015.07.014

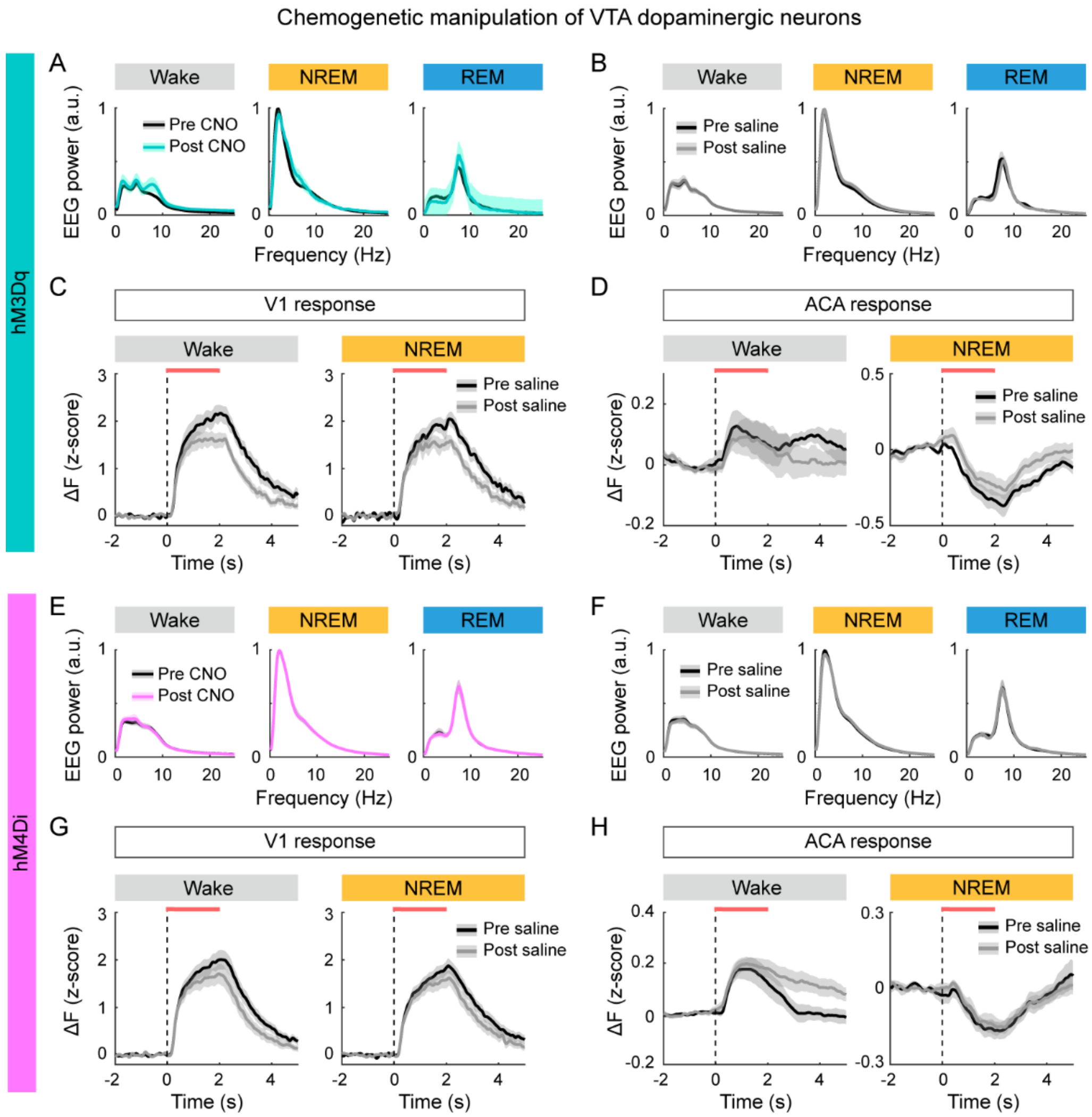

Figure S1. Effects of chemogenetic manipulation of VTA dopaminergic neurons on EEG power spectra, and laser-evoked calcium activity in saline control experiments. (A) EEG power spectra in each brain state before and after CNO injection in mice expressing hM3Dq in VTA dopaminergic neurons (*n* = 6 mice). (B) Similar to (A) but for saline injection. (C) V1 pyramidal neuron calcium activity evoked by optogenetic V1 stimulation before and after saline injection during wakefulness (left) or NREM sleep (right). Baseline activity was subtracted for each trial before averaging. Horizontal red bar, laser stimulation. (D) Similar to (C) but for ACA pyramidal neuron calcium activity. (E-H) Similar to (A-D),

but for mice expressing hM4Di in VTA dopaminergic neurons (*n* = 7 mice). Shading, ± SEM.

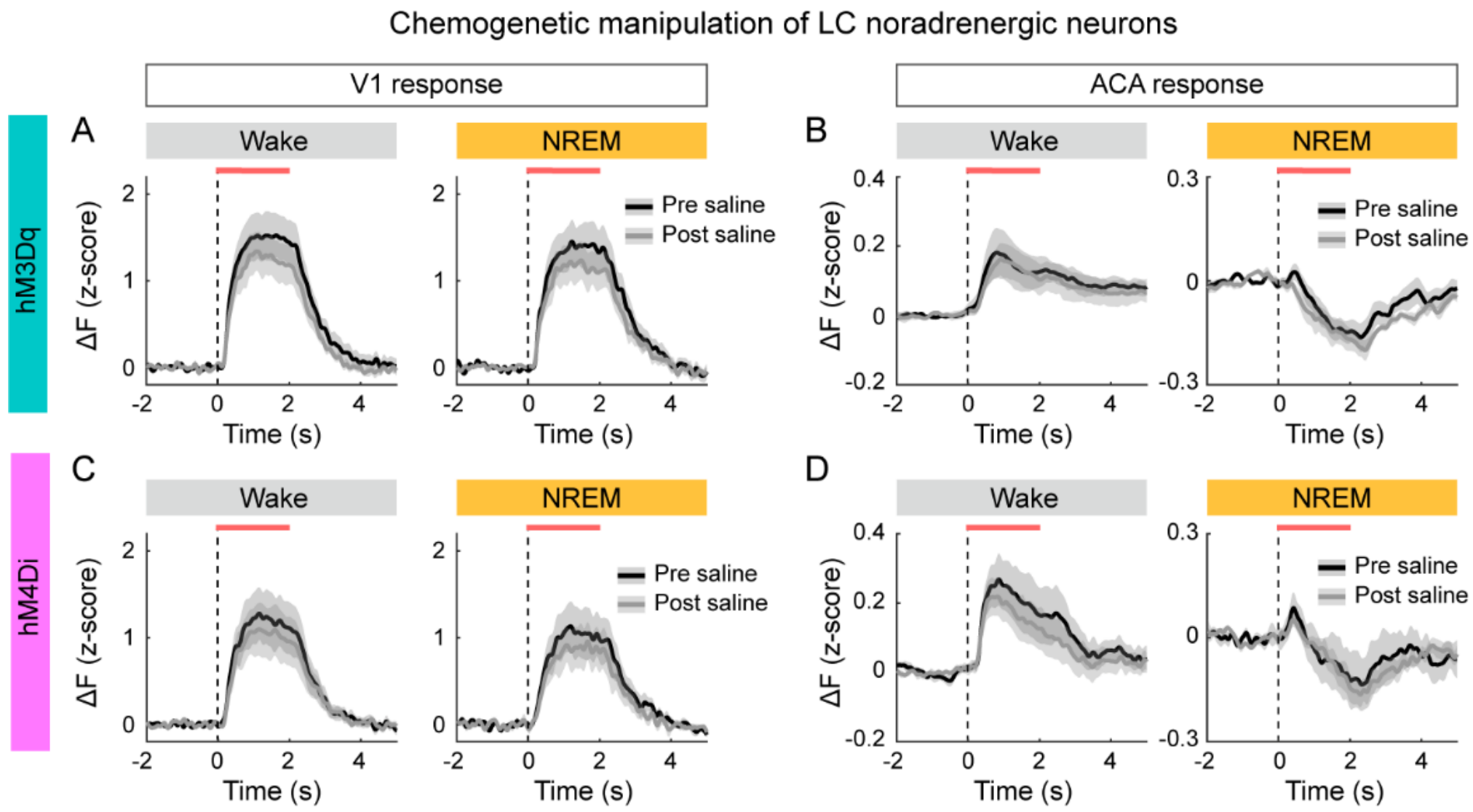

Figure S2. Laser-evoked calcium activity in saline control experiments of chemogenetic manipulation of LC noradrenergic neurons. (A) V1 pyramidal neuron calcium activity evoked by optogenetic V1 stimulation before and after saline injection during wakefulness (left) or NREM sleep (right). Baseline activity was subtracted for each trial before averaging (*n* = 6 mice). Horizontal red bar, laser stimulation. (B) Similar to (A) but for ACA pyramidal neuron calcium activity. (C and D) Similar to (A and B), but for mice expressing hM4Di in LC noradrenergic neurons (*n* = 6 mice). Shading, ± SEM.

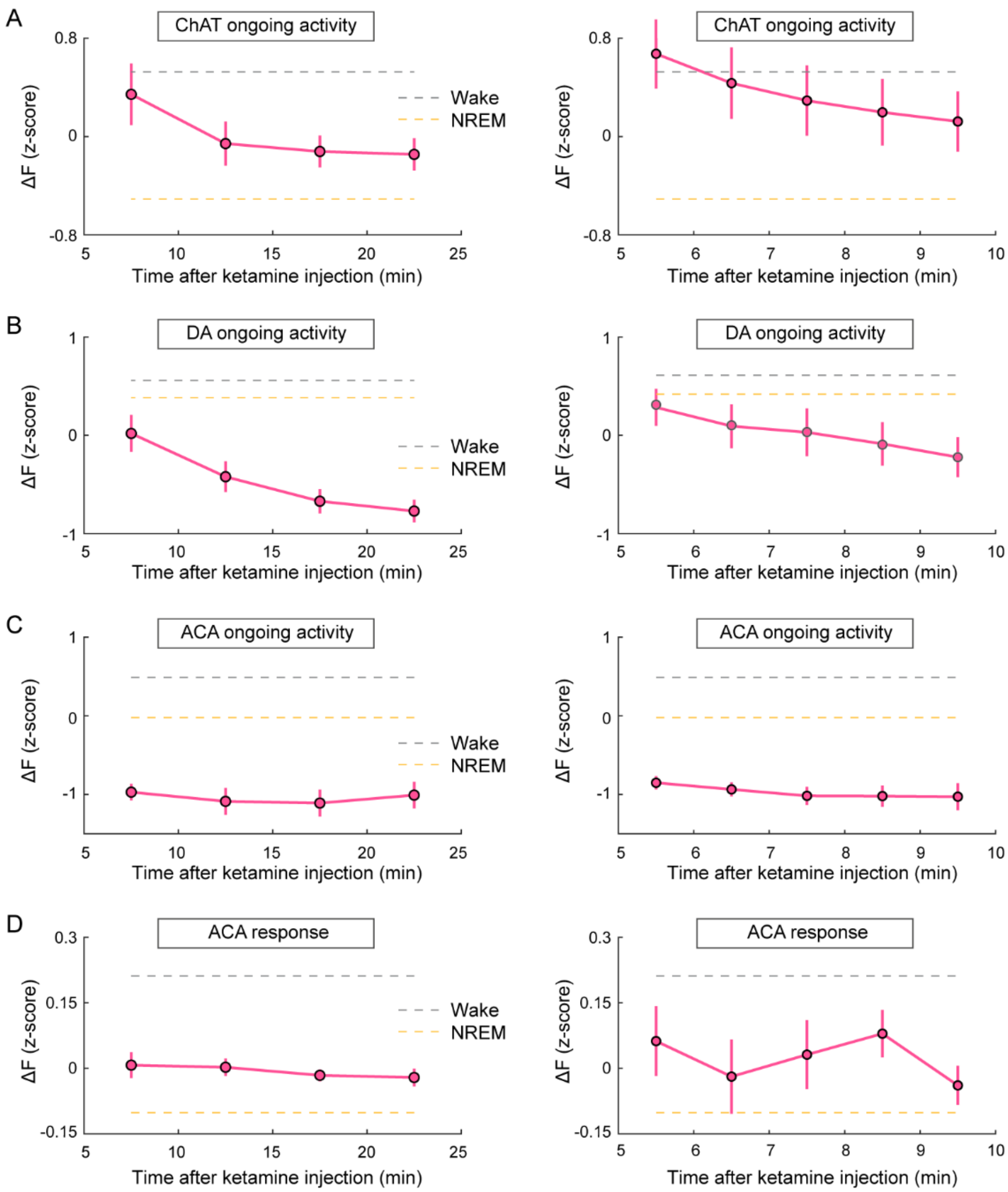

Figure S3. Calcium activity following ketamine injection. (A) The ongoing activity (mean ± SEM) of basal forebrain cholinergic neurons following ketamine injection (n = 6 mice). Data within 5~10 min after ketamine injection are shown on the right. (B) Similar to (A) but for the ongoing activity of VTA dopaminergic neurons (n = 6 mice). (C) Similar to (A) but for the ongoing activity of ACA pyramidal neurons (n = 6 mice). (D) V1-evoked calcium activity in ACA pyramidal neurons following ketamine injection (n = 8 mice). Dashed lines indicate the average calcium activities during wakefulness or NREM sleep

prior to ketamine injection.

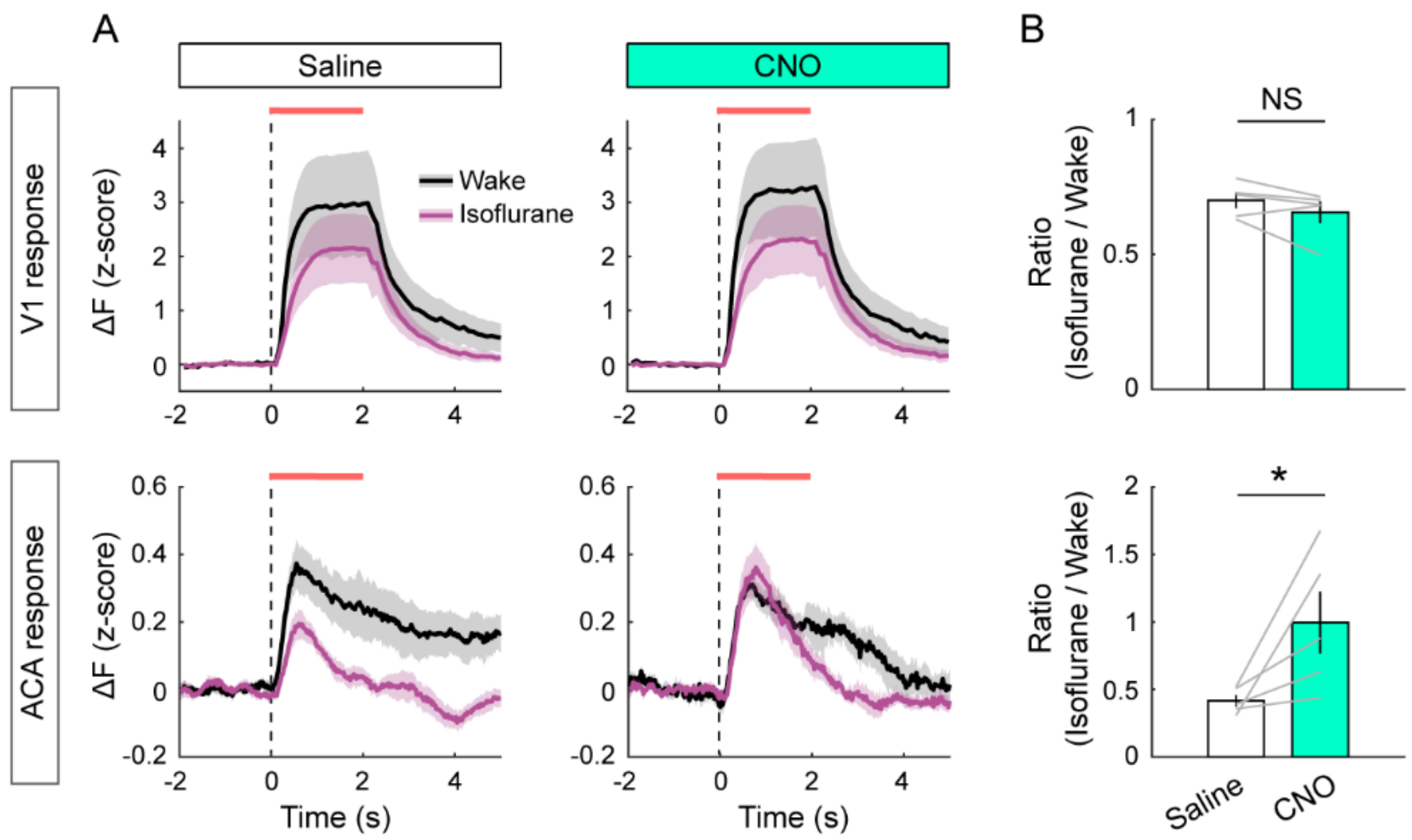

Figure S4. Effects of chemogenetic activation of VTA dopaminergic neurons on V1-to-ACA activity propagation following isoflurane-induced anesthesia. (A) Optogenetically evoked calcium activity in V1 pyramidal neurons (top) during wakefulness or anesthesia. Saline (left) or CNO (1 mg/kg, right) was injected at the beginning of anesthesia. Data were averaged across mice expressing hM3Dq in dopaminergic neurons. Baseline signal was subtracted for each trial before averaging. Horizontal red bar, laser stimulation. (B) Ratio of averaged response amplitudes during anesthesia and during wakefulness in V1 (top) and ACA (bottom). For V1 (top), no significant difference was found. For ACA (bottom), P = 0.04 (n = 5 mice, paired t-test). Bar and error bar, mean ± SEM. Shading, ± SEM. *P < 0.05; NS, not significant.
